# No impact of cyantraniliprole on the hibernation success of bumble bees (*Bombus terrestris audax*) in a soil‐mediated laboratory exposure study

**DOI:** 10.1002/ece3.70328

**Published:** 2024-10-01

**Authors:** Alberto Linguadoca, Morgan A. Morrison, Luca Menaballi, Peter Šima, Mark J. F. Brown

**Affiliations:** ^1^ Department of Biological Sciences Royal Holloway University of London Egham UK; ^2^ Environment, Plants & Ecotoxicology Unit, European Food Safety Authority (EFSA) Parma Italy; ^3^ International Centre for Pesticides and Health Risk Prevention L. Sacco University Hospital Milan Italy; ^4^ Koppert s.r.o. Nové Zámky Slovakia

**Keywords:** hibernation, insecticide, lethal, queen, sub‐lethal, toxicity

## Abstract

Increasing evidence shows that wild bees, including bumble bees, are in decline due to a range of stressors, including pesticides. Our knowledge of pesticide impacts has consequently grown to enable the design of increasingly realistic risk assessment methods. However, one area where knowledge gaps may still hinder our ability to assess the full range of bee‐pesticide interactions is the field of exposure. Exposure has historically been linked to either direct contact with pesticides or the ingestion of contaminated pollen and nectar by bees. However, bumble bees, and other wild bees, may also be exposed to pesticides while using contaminated soil as an overwintering substrate. Yet knowledge of how soil‐mediated exposure affects bumble bee health is lacking. Here we take one of the first steps towards addressing this knowledge gap by designing a method for testing the effects of soil‐mediated pesticide exposure on bumble bee queen hibernation success. We measured hibernation survival, body weight change and abdominal fat content and found that none of these responses were affected by a field realistic soil exposure to the novel insecticide cyantraniliprole. Our study may help in developing a standardised method to test the effects of the soil‐mediated pesticide exposure route in bumble bee queens.

## Graphical abstract

We tested the effects of soil‐mediated, field‐realistic exposure to cyantraniliprole on bumble bee queen hibernation success. We measured survival, body weight change and abdominal fat content of queens and found that none of these responses were affected by the chemical exposure. Our study may help in developing a standardised method to test the effects of the soil‐mediated pesticide exposure route in bumble bee queens.

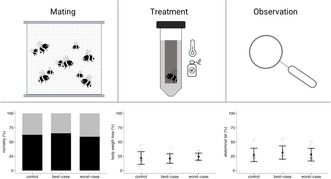

## 1 INTRODUCTION

Wild bees, including bumble bees, are crucial pollinators (IPBES, 2016; Klein et al., 2006). However, multiple anthropogenic stressors are interactively causing the decline of their populations (Potts et al., 2016), with potential knock‐on effects on food system stability (Gallai et al., 2009; Garibaldi et al., 2011) and biodiversity (Biesmeijer et al., 2006).

The growing evidence that pesticides may be pivotal in this paradigm (Siviter et al., 2021) has led to repeated calls to ensure environmental safety of pesticide use prior to licencing (Topping et al., 2020). Risk assessment schemes for bees have consequently evolved towards increasingly elaborate methodologies (EFSA, 2023). However, knowledge gaps still exist that may jeopardise our ability to adequately characterise traits and processes shaping bee vulnerability to pesticides (Schmolke et al., 2021). One example of how fragmentary knowledge may still limit our ability to assess the full range of pesticide–bee interactions, and thus the potential impact of pesticides on bees, is in the field of exposure (Gradish et al., 2019; Sgolastra et al., 2019). Specifically, regulatory science has so far quantitatively linked bee exposure to oral ingestion of contaminated pollen and nectar, or to contact with spray or surface residues (EFSA, 2013; US EPA, 2014a). However, pesticides are known to reach multiple environmental compartments upon application, including soil (Silva et al., 2019). Given that many bee species use soil as a nesting or overwintering substrate (Gradish et al., 2019; Schmolke et al., 2021; Sgolastra et al., 2019), relying on an understanding of plant‐mediated exposure may be insufficient to protect such species from pesticide impacts.

In spite of the evident relevance of this route of exposure, surprisingly few studies have either quantified it (Rondeau, Baert, et al., 2022; Rondeau, Willis Chan, et al., 2022), or characterised its impacts on soil‐dwelling bee species (Main et al., 2020; Rondeau & Raine, 2024a, 2024b; Willis Chan et al., 2019; Willis Chan & Raine, 2021). Specifically, to our knowledge, only one study has so far characterised its hazards in bumble bees, a major group of pollinators in temperate, alpine, and sub‐arctic regions (Rondeau & Raine, 2024b). Both because of these knowledge gaps and due to the historical focus on honey bees, which do not hibernate in or collect soil, as a model species in risk assessment (Franklin & Raine, 2019), regulatory schemes have so far not included a soil‐mediated exposure route (EFSA, 2013; US EPA, 2014a). However, recent evidence describing soil as an important exposure matrix for bees (Gradish et al., 2019) has sparked increased interest in establishing potential links between soil‐mediated exposure and individual and population‐level impacts of pesticides (Christmann, 2022; Fortuin et al., 2021; Rondeau, Baert, et al., 2022; Rondeau & Raine, 2024a, 2024b; Rondeau, Willis Chan, et al., 2022).

Here we take one of the first steps towards addressing this knowledge gap by designing a laboratory methodology for testing the effects of soil‐mediated pesticide exposure on bumble bee queen hibernation success. We used the buff‐tailed bumble bee (*Bombus terrestris*) as a model species, as it is rapidly becoming a reference for regulatory testing of chemicals (OECD, 2017a, 2017b). The colony lifecycle of this species typically lasts 6–7 months (Gurel et al., 2008). Queen and male emergence and mating typically takes place in summer (Gurel et al., 2008), upon which queens undergo overwintering. The first seasonal flights of this species can happen as early as February in continental climate (Goulson, 2003) or November in Mediterranean areas (Gurel et al., 2008), where the centre of the distribution of this species lies (Rasmont et al., 2008). According to Rasmont et al. (2008) colony foundation in *B. terrestris* may also take place in autumn or winter after summer diapause in low‐altitude Mediterranean climates. While the overwintering duration may be highly dependent on climatic conditions (Rasmont et al., 2008), queens of this species typically overwinter in soil cavities for several months before establishing new colonies (Alford, 1969). Therefore, it can be expected that this species may experience long‐term exposure to pesticides via agricultural soil, if contaminated (Rondeau, Baert, et al., 2022; Rondeau, Willis Chan, et al., 2022). Additionally, *B. terrestris* was shown to be particularly vulnerable to pesticide exposure prior to colony founding (Baron et al., 2017), making this species a suitable model candidate for testing the effects of soil‐mediated pesticide exposure.

Here we use the anthranilic diamide insecticide cyantraniliprole (synonym: Cyazypyr™) as our focal pesticide (Selby et al., 2013). Its selection was driven by its recent introduction (European Commission, 2016; US EPA, 2014b), widespread use patterns and likelihood of bee exposure (Rondeau, Baert, et al., 2022; Rondeau, Willis Chan, et al., 2022). Cyantraniliprole targets ryanodine receptors and modulates them to trigger uncontrolled muscular release of calcium ions, which ultimately cause insect pest mortality (Sattelle et al., 2008). Plant protection products containing this active substance are marketed for a wide range of agricultural uses including soil‐directed applications and seed treatments in winter and summer crops (see Section 3.1). Upon application, cyantraniliprole may contaminate agricultural soil with moderate to high persistence (i.e., DT_90_ up to ≈1 year) (EFSA, 2014). The effects of cyantraniliprole on honey bees were investigated in a recent regulatory assessment (EFSA, 2014), which indicated potential risks to honey bees of a subset of the representative uses. The same report included a bumble bee glasshouse exposure study, which showed “potentially treatment‐related” effects on queen, but not worker survival (EFSA, 2014). A recent study estimated the acute oral LD_50_ of cyantraniliprole as >0.54 μg/bee in bumble bee (*Bombus impatiens*) workers (Mundy‐Heisz et al., 2022). Rondeau and Raine (2024b) investigated the impacts of soil‐mediated exposure to cyantraniliprole in hibernating *Bombus impatiens* queens and found no impact on queen survival during or after hibernation. However, the authors reported size‐dependent lethal and sublethal effects of insecticide exposure in bumblebee queens, with more pronounced effects in heavier queens.

Cyantraniliprole was also tested in a honey bee larval test where the acute and chronic LD_50s_ were estimated as 0.047 and 0.064 μg/larva respectively (Kim et al., 2022). In addition, diamide insecticides have been found to interact with the honey bee immune system in a recent transcriptomic study (Liu et al., 2023), suggesting sub‐lethal impacts modulated through the immune system.

Here we test the hypothesis that a long‐term, soil‐mediated, realistic exposure of bumble bee queens to cyantraniliprole affects their overwintering survival, bodyweight change, and abdominal fat stores (as a proxy for immunocompetence) (see Section 2.6).

## 2 MATERIALS AND METHODS

### 2.1 Exposure characterisation

Cyantraniliprole is authorised for soil directed applications, including seed treatments on summer and winter crops at rates up to 449 g a.s./ha (e.g., Fortenza red, 600 g/L cyantraniliprole, FS; EPA registration n. 100‐1418, decision n. 535142, September 22, 2018).

To characterise a field‐realistic exposure regime, we used two complementary approaches. First, we modelled the Predicted Environmental Concentration in soil (PEC_SOIL_) following the indications of the FOrum for Co‐ordination of pesticide fate models and their USe (FOCUS) working group (Boesten et al., 1997). Specifically, we used the following equation:

$${\mathrm{PEC}}_{\text{SOIL}}=\frac{A\cdot \left(1-f_{\mathrm{int}}\right)}{100\cdot d\cdot \mathrm{bd}}$$

where *A* = application rate (g/ha); *f*_int_ = fraction intercepted by the crop canopy; *d* = soil depth (cm) and bd = dry soil bulk density (g/cm^3^).

We modelled a seed treatment application of 449 g a.s./ha, assuming that 100% of the treatment would reach the soil (*f*_int_ = 0). The mean overwintering depth for *B. terrestris* is ≈5 cm (Alford, 1969), which is a realistic sowing depth for crop species (Gruber et al., 2010) and a standard value used in the environmental risk assessment of pesticides (Boesten et al., 1997). Therefore, we assumed cyantraniliprole to homogeneously contaminate the top 5 cm of soil (i.e., *d* = 5). Consistent with the indications of the FOCUS working group, we set a value of 1.5 g/cm^3^ for soil bulk density (Boesten et al., 1997). Using these input parameters, we estimated the initial PEC_SOIL_ as 0.6 mg a.s./kg dry weight.

In the second approach, we used published evidence available at the time of the experiment (Zhang et al., 2019), which measured residues of up to 1.85 mg/kg in soil upon seed treatment of maize with Verimark (cyantraniliprole, 19% SC) at a rate of 2 g a.s./kg seed (≈60 g a.s./ha; Wei Mu, personal communication).

Based on the above, we spiked an artificial soil (see Section 3.2) with the field‐realistic concentrations of 0.6 and 1.85 mg cyantraniliprole/kg dry soil weight. Hereby we refer to these exposure levels as best‐ and worst‐case exposure respectively.

Further discussion on the environmental realism of our exposure levels is reported in the Appendix S1.

### 2.2 Pesticide solutions and exposure medium

A 500 mg a.s./L stock solution was prepared by dissolving cyantraniliprole (purity ≥95.0%, PESTANAL®, Sigma‐Aldrich, UK) in acetone (Sigma‐Aldrich, UK) and subsequently preserving it at −20°C in complete darkness. Water dilutions of the initial stock (cyantraniliprole: 4.3–13.2 ppm; acetone: 0.9%–2.6%) were used to spike an artificial soil (OECD, 2016) at the desired concentrations of 0.6 and 1.85 mg cyantraniliprole/kg dry soil weight. The artificial soil (which was chosen over a natural soil for standardisation purposes) consisted of thoroughly mixed air‐dried, finely ground sphagnum peat (5% w/w, dry weight); kaolin clay (20% w/w, dry weight) and air‐dried industrial sand (74% w/w, dry weight). The spike solution, which was also used to dampen the soil, was thoroughly mixed by hand for ≈5 min into the dry material in a ratio of 0.14:1 (w/w). The total acetone content of the treatments never exceeded 0.37% (w/w) of the soil weight. Therefore, the untreated soil was spiked at the same weight ratio with a pesticide‐free 0.37% (w/w) acetone aqueous solution.

The concentrations of cyantraniliprole in the exposure medium were not analytically verified.

### 2.3 General housing and handling conditions

Eight queen‐right bumble bee (*Bombus terrestris* ssp. *audax*) colonies with sexual brood at all stages of pre‐imaginal development were provided by a commercial supplier (Koppert, SK) in December 2020. Two of these colonies were used for male production, while the remaining 6 boxes were used to source unmated queens (known as gynes). Upon delivery, bees were kept at 25°C and ≈50% relative humidity in darkness and manipulated under red light. Upon emergence, gynes and males were sorted by sex and colony of origin into empty colony boxes, which were filled with a layer of cat litter to absorb excess moisture. Consistent with standardised testing methods with bumble bees (OECD, 2017a, 2017b), particularly small or very large gynes were excluded upon visual inspection of the colonies. Bees were fed inverted sugar syrup (Koppert, SK), until they were transferred into the brood‐less boxes, when they were given ad libitum access to 30% (w/w) sucrose via gravity feeders. All colony boxes were also given a daily provision of ≈15 g fresh‐frozen, honey bee‐collected pollen pellets (Agralan, UK).

### 2.4 Mating

Mating took place in wooden arenas (0.6 × 0.5 × 0.5 m) with plastic mesh walls at ≈21°C under natural light (Baron et al., 2017). Each mating round consisted of 2 h, during which unrelated gynes and males were constantly monitored for mating. Males older than 7 days of age and 18–24‐day‐old gynes were used in a ratio of 2:1.

Given the long lifespan of *B. terrestris* queens, we consider unlikely that the age range of queens at mating may have significantly impacted survival during hibernation in our experiment (Amin et al., 2007).

The maximum initial number of bees per arena was 50 males and 25 gynes. Mating pairs were promptly isolated into cages made from upside down, lidded, clear plastic cups (10 cm tall, 9 cm diameter at base, 6 cm diameter at top). Isolated queens were then moved to a dark rearing room at 25°C and ≈50% relative humidity for a 3‐day acclimatisation period, during which they were fed fresh frozen loose pollen and 30% (w/w) sucrose. A total of 134 queens were mated, 6 of which died during acclimatisation.

### 2.5 Hibernation

At the end of acclimatisation, queens were weighed to the nearest milligram (Scout® STX, Ohaus, UK) and transferred individually into hibernation units (See Figure S1). These consisted of 50 mL centrifuge tubes (Sarstedt, UK) with 2 mm ventilation holes (*n* = 3) drilled into the lid. Each tube was filled with ≈40 mL artificial soil, in which we included a ≈ 1.5 cm wide hole dug lengthwise, to maximise contact exposure. Individually housed queens were kept in a dark incubator at 4°C for 67 days, after which mortality and bodyweight were recorded. This hibernation period was considered representative of the lower end of the distribution of the possible diapause durations for our model species. Furthermore, it was chosen given uncertainty around background mortality under these conditions, and previous studies showing variable but sometimes high (up to 48%) mortality between 2 and 3 months under more pristine conditions (Brown et al., 2003). A pilot study (results not shown) suggested that queens may experience non‐negligible mortality levels immediately after hibernation. Therefore, live queens post‐hibernation were then individually transferred into plastic cages (Nicot, Nicotplast, FR) and kept at the same conditions described under Section 3.3 for 2 additional days, during which post‐hibernation mortality was recorded. During this period, bees were fed 30% sucrose through a 5 mL syringe (BD emerald, UK) with the tip removed. All bees were later stored at −20°C for abdominal fat extraction and size measurement (Mitutoyo, UK), using the inter‐tegular span as a proxy for bee size (Cane, 1987).

We defined hibernation mortality as the cumulative proportion of dead bees during hibernation and post‐hibernation. Bodyweight loss was defined as the bodyweight change before and after hibernation normalised by the bodyweight before hibernation.

### 2.6 Abdominal fat extraction

We considered fat body mass as a proxy for immunocompetence of individual bees (Korner & Schmid‐Hempel, 2005; Vanderplanck et al., 2021). Following Brown et al. (2000) and Gekière et al. (2022), isolated queen abdomens were dried at 70°C for 3 days before being weighed to the nearest milligram. Abdomens were then transferred into laboratory glass vials filled with 4 mL diethyl ether (Sigma‐Aldrich, UK) for 24 h before being rinsed twice with the same solvent. The abdomens were dried again at 70°C for 7 days and then weighed one final time. The abdominal fat stores were defined as the dry abdominal weight difference before and after extraction divided by the dry abdominal weight before extraction.

### 2.7 Statistical analysis

We used an information‐theoretic approach based on AIC_c_. For each analysis, we included a null model, a full model and all biologically meaningful subsets of the full model. We then averaged all models, including the null model within a 95% confidence set, defined by the AIC_c_‐based weights of the individual models.

Survival was analysed using a generalised mixed effect model with a binomial distribution and logit link function, with treatment, male colony of origin and their interaction as fixed effects and female colony of origin as a random effect. An additional survival analysis including bodyweight as a fixed effect is included in the Appendix S1.

Weight loss and abdominal fat stores were analysed using a linear model with treatment, size and their interaction as fixed effects. Details on the model selection and parameter estimates (±95% confidence intervals) are provided in the Tables S2 and S3. All analyses were carried out in ‘R' programming software (R Core Team, 2022) version 4.2.2 using the packages “ggplot2” (Wickham, 2009), “dplyr” (Wickham et al., 2022), “lme4” (Bates et al., 2015) and “MuMIn” (Bartoń, 2022).

## 3 RESULTS

### 3.1 Survival

**FIGURE 1:**
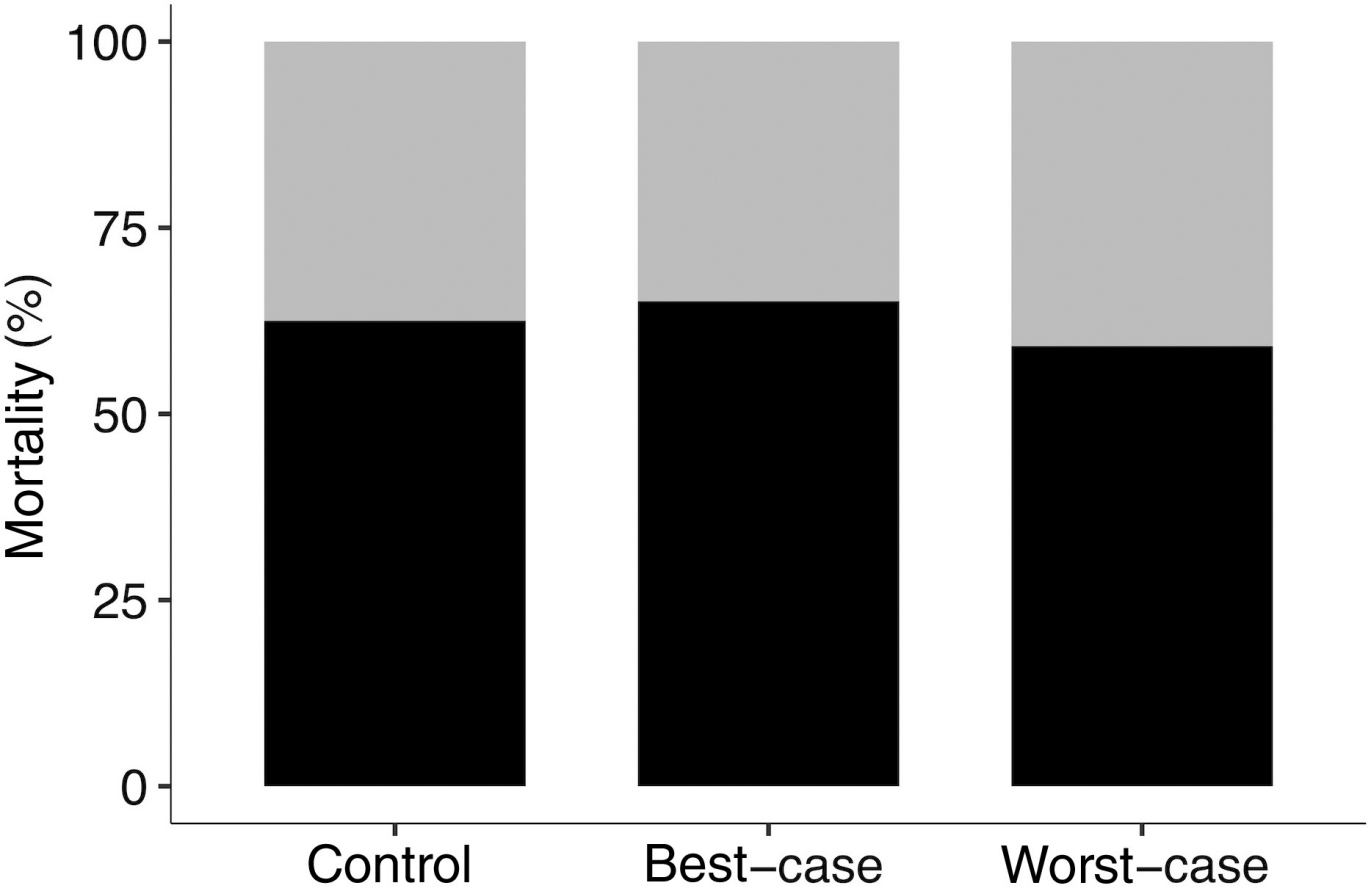
Bar chart of the proportion of dead (black) and live (grey) queens at the end of the post‐hibernation observation phase. *N* = 127 (*n*; control: 40, best‐case: 43, worst‐case: 44).

The total number of queens before hibernation was 127(control: 40, best‐case: 43, worst‐case: 44), 80 of which (control: 25, best‐case: 28, worst‐case: 26) survived the post‐hibernation observation phase. The cumulative mortality of queens across treatments at the end of the post‐hibernation observation phase (Figure 1; control: 63%, best‐case: 65%, worst‐case: 59%) was not affected by treatment (best‐case, Parameter Estimate (PE) = 0.01, 95% Confidence Interval (CI) = −0.33 to 0.35; worst‐case, PE = −0.03, 95% CI = −0.40 to 0.34) or male colony of origin (colony (ID:14): PE = −0.69, 95% CI = −1.73 to 0.34).

### 3.2 Weight loss

**FIGURE 2:**
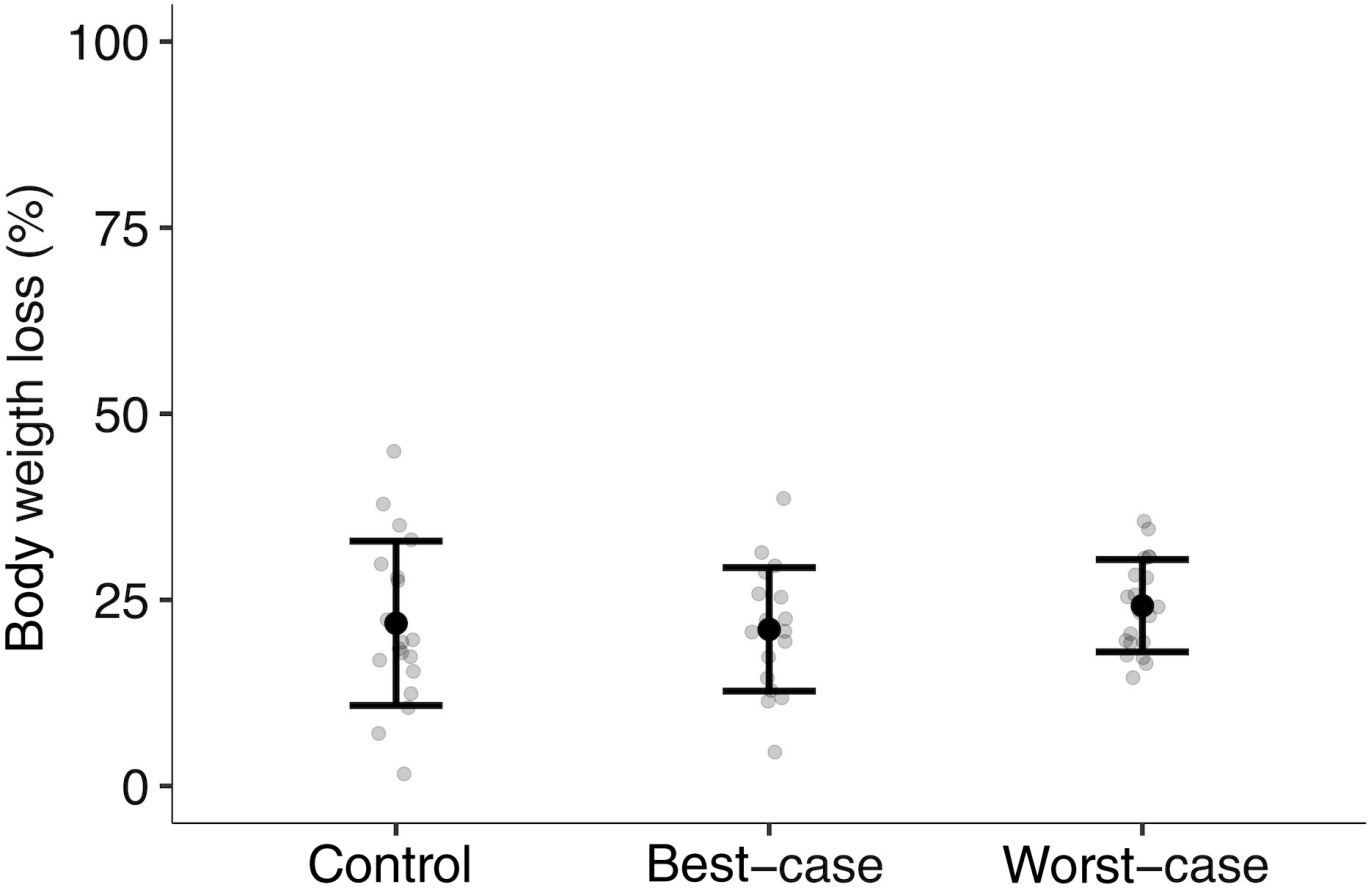
The mean (±SD) body weight loss of the live queens at the end of the hibernation phase. *N* = 57 (*n*; control: 19, best‐case: 18, worst‐case: 20).

The average queen weight before hibernation was 0.81 g (control: 0.82 g, best‐case: 0.82 g, worst‐case: 0.80 g; Table S1), while the average weight of the live queens after the post‐hibernation observation phase was 0.65 g (control: 0.63 g, best‐case: 0.67 g, worst‐case: 0.64 g; Table S1). Live queens at the end of the post‐hibernation observation phase lost on average 22.42% of their bodyweight (Figure 2; control: 21.86%, best‐case: 21.04%, worst‐case: 24.21%).

There was no significant effect of treatment (best‐case, PE = −10.77, 95% CI = −109.46 to 87.92; worst‐case, PE = −5.57, 95% CI = 63.24 to 52.1), size (PE = −2.74, 95% CI = −13.83 to 8.35) or their interaction (best‐case, PE = −1.71, 95% CI = −14.08 to 17.5; worst‐case, PE = 0.96, 95% CI = −8.44 to 10.35), on the bodyweight loss of the live queens after hibernation.

### 3.3 Abdominal fat stores

**FIGURE 3:**
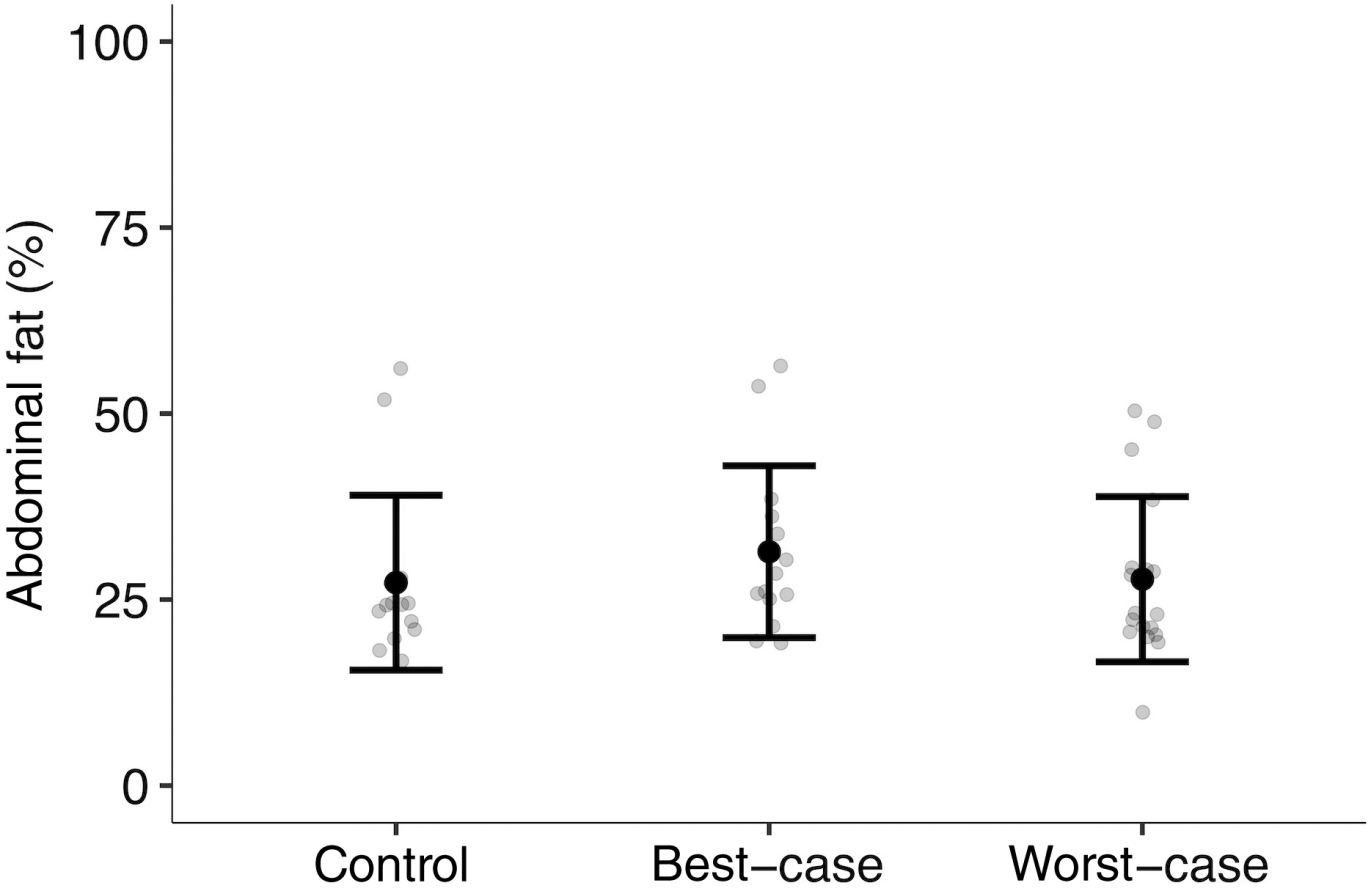
The mean (± SD) fat body stores of the live queens at the end of the post‐hibernation phase. *N* = 46 (*n*; control: 14, best‐case: 14, worst‐case: 18).

The average abdominal fat weight of the live queens after hibernation was 45.94 mg (control: 46.4 mg, best‐case: 48.5 mg, worst‐case: 43.6 mg; Table S1), corresponding to 28.72% of the dry abdominal stores (Figure 3; control: 27.27%, best‐case: 31.43%, worst‐case: 27.74%; Table S1).

There was no significant effect of treatment (best‐case, PE = 0.64, 95% CI = −3.83 to 5.11; worst‐case, PE = 0.01, 95% CI = −3.17 to 5.20) or size (PE = −7.35, 95% CI = −23.25 to 856.22) on the abdominal fat stores of the live queens after hibernation.

## 4 DISCUSSION

Using a methodology informed by existing OECD protocols (OECD, 2016, 2017a, 2017b), we found no effects of field‐realistic cyantraniliprole exposure on a key life history stage in bumble bees. Hibernation survival, and weight loss during hibernation of bumble bee queens were unaffected by either high or low exposure to this widely used pesticide.

Cyantraniliprole is labelled as “highly toxic to bees” in the US (EPA registration n. 100–1418, decision n. 535,142), while specific provisions highlighting its risk to “bees and bumble bees” are recommended in the EU (European Commission, 2016). Yet, surprisingly few studies (Kim et al., 2022; Mundy‐Heisz et al., 2022; Rondeau & Raine, 2024b) have so far characterised hazards or risks of cyantraniliprole to bees beyond the evidence already presented in regulatory assessment reports (EFSA, 2014). Our study found no evidence of impacts on queen hibernation survival, bodyweight change and abdominal fat stores. Interestingly, a recent study (Rondeau, Baert, et al., 2022; Rondeau, Willis Chan, et al., 2022) quantified soil contamination of bumble bee hibernation sites in apple orchards and detected cyantraniliprole in 65% of the sampled soils at a maximum concentration of 0.15 mg a.s./kg. Our exposure concentrations are 4 to 13 times higher than the ones detected in this study, suggesting that, at least in orchard sites, it is unlikely that this pesticide would have negative impacts on hibernation survival in the field.

However, we note that in orchards cyantraniliprole is most likely applied via foliar sprays, which may explain the lower potential exposure relative to the exposure concentrations we used in our study. The concentrations we used were based on field‐realistic levels in non‐orchard crops, and so still provide evidence for a lack of effect in broader agricultural systems. Furthermore, there may be scenarios where soils are contaminated at higher concentrations than the ones tested in our study (see Appendix S1). For these circumstances, the risks for hibernating bumble bee queens remains unknown.

Our results are coherent with a recent study by Rondeau and Raine (2024b), where bumble bee (*Bombus impatiens*) queens were exposed to cyantraniliprole for 7.5 months via pesticide‐spiked natural soil in a laboratory hibernation setup. The field‐realistic exposure of 16 μg/kg used by Rondeau and Raine (2024b) was lower than the exposure used in in our experiment. In contrast, the authors aimed to mimic a worst‐case diapause duration in the laboratory, resulting in a substantially longer potential period of exposure than in our experiment. Rondeau and Raine (2024b) found no effect of the soil‐mediated exposure to cyantraniliprole on queen hibernation survival and bodyweight change. However, they reported size‐dependent effects (i.e., proportional to bodyweight) of cyantraniliprole on post‐hibernation survival, colony initiation and reproductive parameters. The species and exposure scenarios tested in our study are not fully comparable with Rondeau and Raine (2024b). Nevertheless, our results provide additional evidence for a lack of impacts on hibernation success at higher exposure levels (albeit for shorter duration) than the ones tested by Rondeau and Raine (2024b). Together the two studies highlight the importance of assessing longer‐term lethal and sublethal impacts of soil‐mediated pesticide exposure beyond those explored in our experiment.

While bumble bee queens may be exposed to concerning levels of pesticides when hibernating in agricultural soil cavities (Rondeau, Baert, et al., 2022; Rondeau, Willis Chan, et al., 2022; Silva et al., 2019), there are no standardised methodologies to assess the impacts of this exposure route. Here we took one of the first steps towards designing a methodology for investigating pesticide toxicity in hibernating queens exposed via artificial soil. For the first time in bee toxicology, we applied a fate modelling approach for the prediction of exposure concentrations in soil, based on pesticide use data. This method, which aims to provide worst‐case realistic estimates (Boesten et al., 1997), was proven in our study to give reasonably accurate predictions of potential exposure in the field (i.e., those measured in Zhang et al., 2019).

However, background levels of mortality were high in our experiment, and significantly higher than might be expected from previous experimental studies of hibernation (Brown et al., 2003). We hypothesise that such mortality was caused by the substrate used during the laboratory hibernation, which might require development to better suit the model species in our experiment. Indeed, sterilised dampened sand has been shown to result in control mortality at levels below 10% in a similar experimental design over a longer hibernation duration than the one tested in our study (Morrison et al., under review). Therefore, we suggest that soil sterilisation will reduce background mortality of control bees to levels more appropriate for a standardised ecotoxicological protocol for future use in risk assessment. Interestingly, weight loss of surviving queens mapped more closely onto what would be expected from previous studies (e.g., Brown et al., 2003), suggesting that if mortality can be reduced this would be a realistic protocol for future investigations of pesticide impacts during hibernation.

Another limitation of our experimental design is that our knowledge of pesticide toxicokinetics by bees is limited and case‐specific (Zaworra et al., 2019). This means that our knowledge of the rates of pesticide absorption, metabolism and elimination of cyantraniliprole by queens hibernating in contaminated soil is limited. Future studies should characterise how soil properties and chemistry of active substances may interactively modulate pesticide uptake, metabolism and elimination in hibernating queens. We note that there is evidence for a reduction in the abundance of a putative detoxification enzyme during late diapause (Colgan et al., 2019), which would predict potentially higher impacts of active substances during hibernation, in contrast to the results we found here. Furthermore, how different diapause durations might modulate pesticide toxicity deserve exploration.

In addition, the development of dose–response designs for a subset of substances known to drive lethal and sublethal effects would provide candidates for positive controls (OECD, 1998) and ultimately aid the interpretation of null or positive results across tests.

In conclusion, understanding the impacts of pesticide exposure through soils is a key open question in pollinator conservation. Our study may provide a good basis to develop a standardised test methodology to characterise hazards of soil‐mediated pesticide exposure in bumble bee queens.

## AUTHOR CONTRIBUTIONS

**Alberto Linguadoca:** Conceptualization (lead); data curation (lead); formal analysis (lead); investigation (lead); methodology (lead); visualization (lead); writing – original draft (lead); writing – review and editing (equal). **Morgan A. Morrison:** Conceptualization (equal); investigation (equal); methodology (supporting); writing – review and editing (equal). **Luca Menaballi:** Methodology (supporting); writing – review and editing (equal). **Peter Šima:** Resources (equal); writing – review and editing (equal). **Mark J. F. Brown:** Conceptualization (lead); funding acquisition (lead); investigation (supporting); methodology (equal); supervision (lead); writing – review and editing (lead).

## CONFLICT OF INTEREST STATEMENT

The authors declare no competing interests. The views expressed in this article are the authors only and do not necessarily reflect the views, position or scientific work of the European Food Safety Authority.

## Supporting information


Data S1

## Data Availability

All data are available from the Zenodo repository: https://doi.org/10.5281/zenodo.7649989.
