# Supplementary material for: No impact of cyantraniliprole on the hibernation success of bumble bees (Bombus terrestris audax) in a soil‐mediated laboratory exposure study
Source: Ecol Evol. 2024 Oct 1;14(10):e70328. doi: 10.1002/ece3.70328 (PMC11445450; doi:10.1002/ece3.70328)
Supplement: Supplementary file 1 — Data S1 [file ECE3-14-e70328-s001.docx]

**Supplementary material**

**No impact of cyantraniliprole on the hibernation success of bumble bees (*Bombus terrestris audax*) in a soil-mediated laboratory exposure study**

Alberto Linguadoca^1,2^*, Morgan A. Morrison^1^, Luca Menaballi^3^, Peter Šima^4^, Mark J. F. Brown^1^

^1^Department of Biological Sciences, Royal Holloway University of London, Egham, UK

^2^ Pesticide Peer Review Unit, European Food Safety Authority (EFSA), Parma, Italy

^3^ International Centre for Pesticide and Health Prevention, L. Sacco University Hospital, Milano, Italy

^4^ Koppert s.r.o., Nové Zámky, Slovakia

**Corresponding author (*[*Alberto.Linguadoca.2018@live.rhul.ac.uk*](mailto:Alberto.Linguadoca.2018@live.rhul.ac.uk)*)*

**Contents**

[**Descriptive statistics** 3](#_Toc177978579)

[**Model selection** 5](#_Toc177978580)

[**Full output of the statistical models** 6](#_Toc177978581)

[**Overwintering survival: additional analysis** 8](#_Toc177978582)

[**Exposure realism** 10](#_Toc177978583)

[**References** 14](#_Toc177978584)

# **Descriptive statistics**

Table S1: Summary statistics (sample size, mean, SD, and range) for queen “abdominal fat”, “fresh body weight before hibernation”, “fresh bodyweight post- hibernation”.

| **response variable** | **treatment** | **status** | **sample size (n.)** | **mean (mg)** | **SD (mg)** | **min (mg)** | **max (mg)** |
| --- | --- | --- | --- | --- | --- | --- | --- |
| abdominal fat | control | all | 21 | 30.9 | 87.1 | 51.3 | 15 |
|  |  | alive end | 14 | 46.4 | 13.4 | 30.9 | 76.4 |
|  |  | dead post-hibernation | 4 | 56.8 | 11.2 | 44.7 | 71.3 |
|  |  | dead hibernation | 3 | 66.7 | 17.7 | 54.9 | 87.1 |
|  | best-case | all | 25 | 25.6 | 92.4 | 48.2 | 15.2 |
|  |  | alive end | 14 | 48.5 | 12.3 | 26.9 | 70 |
|  |  | dead post-hibernation | 3 | 52 | 17.1 | 37.2 | 70.7 |
|  |  | dead hibernation | 8 | 46.2 | 20.4 | 25.6 | 92.4 |
|  | worst-case | all | 25 | 19.5 | 96 | 45.5 | 15.7 |
|  |  | alive end | 18 | 43.6 | 10.7 | 19.5 | 67.5 |
|  |  | dead post-hibernation | 4 | 52 | 29.4 | 34.2 | 96 |
|  |  | dead hibernation | 3 | 48.3 | 23.8 | 34.1 | 75.7 |
| fresh body weight before hibernation | control | all | 40 | 820.4 | 109 | 577 | 1090 |
|  | best-case | all | 43 | 819.3 | 88.7 | 581 | 977 |
|  | worst-case | all | 44 | 803.3 | 105.2 | 540 | 982 |
| fresh bodyweight post- hibernation | control | all | 22 | 501 | 849 | 637 | 86.4 |
|  |  | alive end | 15 | 630.5 | 89.4 | 501 | 849 |
|  |  | dead post-hibernation | 4 | 603.8 | 57.8 | 525 | 659 |
|  |  | dead hibernation | 3 | 713.7 | 77.8 | 661 | 803 |
|  | best-case | all | 26 | 488 | 867 | 669.9 | 87.3 |
|  |  | alive end | 15 | 665.9 | 79.4 | 556 | 825 |
|  |  | dead post-hibernation | 3 | 618.7 | 136.3 | 488 | 760 |
|  |  | dead hibernation | 8 | 696.6 | 85.7 | 606 | 867 |
|  | worst-case | all | 25 | 518 | 774 | 654.1 | 68.2 |
|  |  | alive end | 18 | 638.8 | 59.9 | 518 | 731 |
|  |  | dead post-hibernation | 4 | 660.3 | 85.2 | 542 | 744 |
|  |  | dead hibernation | 3 | 737.3 | 43 | 690 | 774 |

# **Model selection**

Table S2: details on the model selection. Averaged models are underlined.

| **response variable** | **model** | **AICc** | **ΔAICc** | **weight** |
| --- | --- | --- | --- | --- |
| survival | treatment + colony (male) + treatment:colony(male) + (1\|colony (female)) | 158 | 8.12 | 0.011 |
|  | treatment + colony (male) + (1\|colony (female)) | 153.6 | 3.7 | 0.102 |
|  | treatment + (1\|colony (female)) | 155.8 | 5.95 | 0.033 |
|  | colony (male) + (1\|colony (female)) | 149.9 | 0 | 0.648 |
|  | null model | 152.2 | 2.3 | 0.206 |
| weight loss (%) | treatment + size + treatment:size | 415.6 | 4.52 | 0.051 |
|  | treatment + size | 415.6 | 4.58 | 0.05 |
|  | treatment | 414.2 | 3.11 | 0.104 |
|  | size | 412 | 0.99 | 0.301 |
|  | null model | 411 | 0 | 0.494 |
| abdominal fat stores (% of dry abdominal weight) | treatment + size + treatment:size | 364.7 | 8.37 | 0.008 |
|  | treatment + size | 359.7 | 3.38 | 0.093 |
|  | treatment | 360.6 | 4.31 | 0.059 |
|  | size | 356.3 | 0 | 0.505 |
|  | null model | 357.2 | 0.82 | 0.335 |

# **Full output of the statistical models**

Table S3: output of the statistical models.

| **Survival** | | | | | | |
| --- | --- | --- | --- | --- | --- | --- |
|  | **Parameter estimate** | **LL CI** | **UL CI** | **Standard Error** | **z value** | **p-value** |
| intercept | 2.6225 | -0.0931913 | 5.3381838 | 1.37238 | 1.893 | 0.0584 |
| colony (male) n. 14 | -0.69319 | -1.7253977 | 0.3390121 | 0.52395 | 1.316 | 0.1881 |
| treatment (best-case) | 0.01157 | -0.3290544 | 0.3522003 | 0.17213 | 0.067 | 0.9469 |
| treatment (worst-case) | -0.02922 | -0.4016664 | 0.3432274 | 0.18852 | 0.154 | 0.8778 |
| **weight loss (%)** | | | | | | |
|  | **Parameter estimate** | **LL CI** | **UL CI** | **Standard Error** | **z value** | **p-value** |
| intercept | 39.3662 | -29.50995 | 108.242449 | 34.8336 | 1.12 | 0.263 |
| size | -2.742 | -13.8305 | 8.346486 | 5.6081 | 0.485 | 0.628 |
| treatment (best-case) | -10.7704 | -109.46498 | 87.924265 | 50.1011 | 0.214 | 0.831 |
| treatment (worst-case) | -5.5735 | -63.24323 | 52.096304 | 29.2098 | 0.189 | 0.85 |
| size:treatment (best-case) | 1.7074 | -14.083 | 17.49776 | 8.0157 | 0.212 | 0.832 |
| size:treatment (worst-case) | 0.9579 | -8.43605 | 10.351922 | 4.7589 | 0.2 | 0.842 |
| **Abdominal fat stores (% of dry abdominal weight)** | | | | | | |
|  | **Parameter estimate** | **LL CI** | **UL CI** | **Standard Error** | **z value** | **p-value** |
| intercept | 74.05756 | -24.665841 | 172.780954 | 49.72992 | 1.47 | 0.141 |
| size | -7.34567 | -23.2535 | 856.22% | 8.01347 | 0.905 | 0.365 |
| treatment (best-case) | 0.63994 | -3.831902 | 5.111776 | 2.24508 | 0.28 | 0.779 |
| treatment (worst-case) | 0.01185 | -3.172191 | 3.195889 | 1.57846 | 0.007 | 0.994 |

**Supplementary figures**


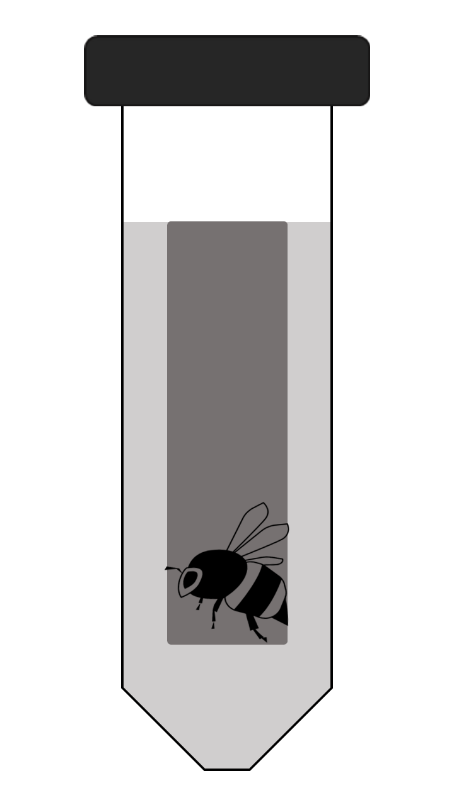


Figure S1: a schematic representation of the overwintering tube setup.

# **Overwintering survival: additional analysis**

During the peer review process it was questioned whether bee weight and its interaction with treatment may have influenced overwintering survival. Therefore, the results of the additional analysis are reported below.

We used the same model selection procedure described in section 2.7 of the main manuscript. Survival was initially analysed using a generalised mixed effect model with a binomial distribution and logit link function, with treatment, male colony of origin, and weight as a fixed effect and queen colony of origin as random effect. However, due to overfitting issues, the random effect was dropped. Consequently, we used a mixed effect model with a binomial distribution and logit link function, with treatment, male colony of origin, weight and queen colony of origin as fixed effects. When the interaction between weight and treatment was included in the model, we observed unrealistic parameter estimates which did not match the observations. Therefore, no interaction was finally included in the model.

We did not find an effect of treatment, weight, or colony of origin (male or female) on overwintering queen survival (Table S4).

We observed a trend towards lower mortality rates in heavier queens (Table S4). Unexpectedly, we did not find statistical confirmation that queen size influenced hibernation survival. A possible explanation for this finding is that statistical detection of bodyweight effects would require larger samples sizes than in our experiment, given the measured variation in queen weights. Indeed, considering that particularly small or very large gynes were excluded from our experiment (see section 2.3 of the main manuscript), the measurable effect size in our experiment may have been narrowed down. Another explanation is that longer diapause durations than in our experiment may result in larger effects of body size, making them more easily detectable effect sizes.

Table S4: the output of the revised survival model

|  | **Parameter estimate** | **LL CI** | **UL CI** | **Standard Error** | **z value** | **p-value** |
| --- | --- | --- | --- | --- | --- | --- |
| (Intercept) | 2.27 | -2.03 | 6.57 | 2.18 | 1.03 | 0.30 |
| Colony (male) 14 | -0.64 | -1.73 | -0.01 | 0.53 | 1.19 | 0.24 |
| Colony (female) 2 | 18.64 | -3808.58 | 3845.86 | 1933.00 | 0.01 | 0.99 |
| Colony (female) 3 | 0.78 | -0.69 | 2.26 | 0.75 | 1.04 | 0.30 |
| Colony (female) 4 | -0.89 | -3.51 | 1.73 | 1.32 | 0.67 | 0.51 |
| Colony (female) 6 | 18.65 | -5530.74 | 5568.03 | 2803.00 | 0.01 | 1.00 |
| Colony (female) 8 | 18.79 | -3351.16 | 3388.75 | 1702.00 | 0.01 | 0.99 |
| Weight pre hibernation | -2.41 | -8.57 | 0.69 | 2.65 | 0.91 | 0.37 |
| Treatment: best-case | 0.01 | -0.91 | 1.18 | 0.13 | 0.06 | 0.95 |
| Treatment: worst-case | -0.02 | -1.40 | 0.71 | 0.15 | 0.13 | 0.90 |

# **Exposure realism**

During the peer review process, it was requested to better justify the choice of the two exposure concentrations used in our study (best-case, predicted: 0.6 mg a.s./kg soil and worst-case, measured: 1.85 mg a.s./kg soil).

Therefore, we carried out a targeted literature search using 2 search strings: i) “cyantraniliprole residue* soil” and ii) “cyantraniliprole concentration* soil”.

Papers were considered relevant whenever they included analytical measurements of cyantraniliprole in soil. From the set of relevant experiments, we extracted the maximum value of the cyantraniliprole in soil and reported it in Table S5 and Figure S2.

The dataset mainly consisted of field residue trials, where the application methods (Figure S2 - B) included seed dressing (n=1), soil spray (n=3) and foliar spray (n=2). Furthermore, one field monitoring study was available (Rondeau, 2024), specifically targeting bumblebee hibernation sites.

All residue measurements were substantially lower than our worst-case exposure concentration (Figure S2 – A), except one (Kandil et al., 2023). However, the application rate per hectare in Kandil et al. (2023) is unknown and may be substantially higher than in the other studies. Considering that this value is clearly outside the distribution of the other datapoints, we considered it a possible outlier.

Overall, we believe that the literature search confirmed the realism of our exposure conditions.

Finally, we acknowledge that the difference between the predicted and measured exposure values in our study is not negligible. A possible explanation for this difference is due to the behaviour of the active substance in the topsoil. Indeed, a reasonable simplification introduced by Boesten et al. (1997) in the estimation of soil concentration via modelling (see section 2.1 of the main manuscript) is that pesticide active substances have uniform distribution within the mixing depth upon exposure. This assumption is likely biased towards the assessment of spray uses, which are by far the most common. However, it is possible that for applications such as seed treatments uniform distribution is not immediately achieved.

Zhang et al. (2019) performed soil sampling closer to the emerging seedling, where residue levels may be higher than between furrows. It could be assumed that with a sufficiently high level of replication of soil samples and an appropriate randomisation of sampling depths/locations and methodologies a lower exposure level would have been measured in the field by Zhang et al. (2019).


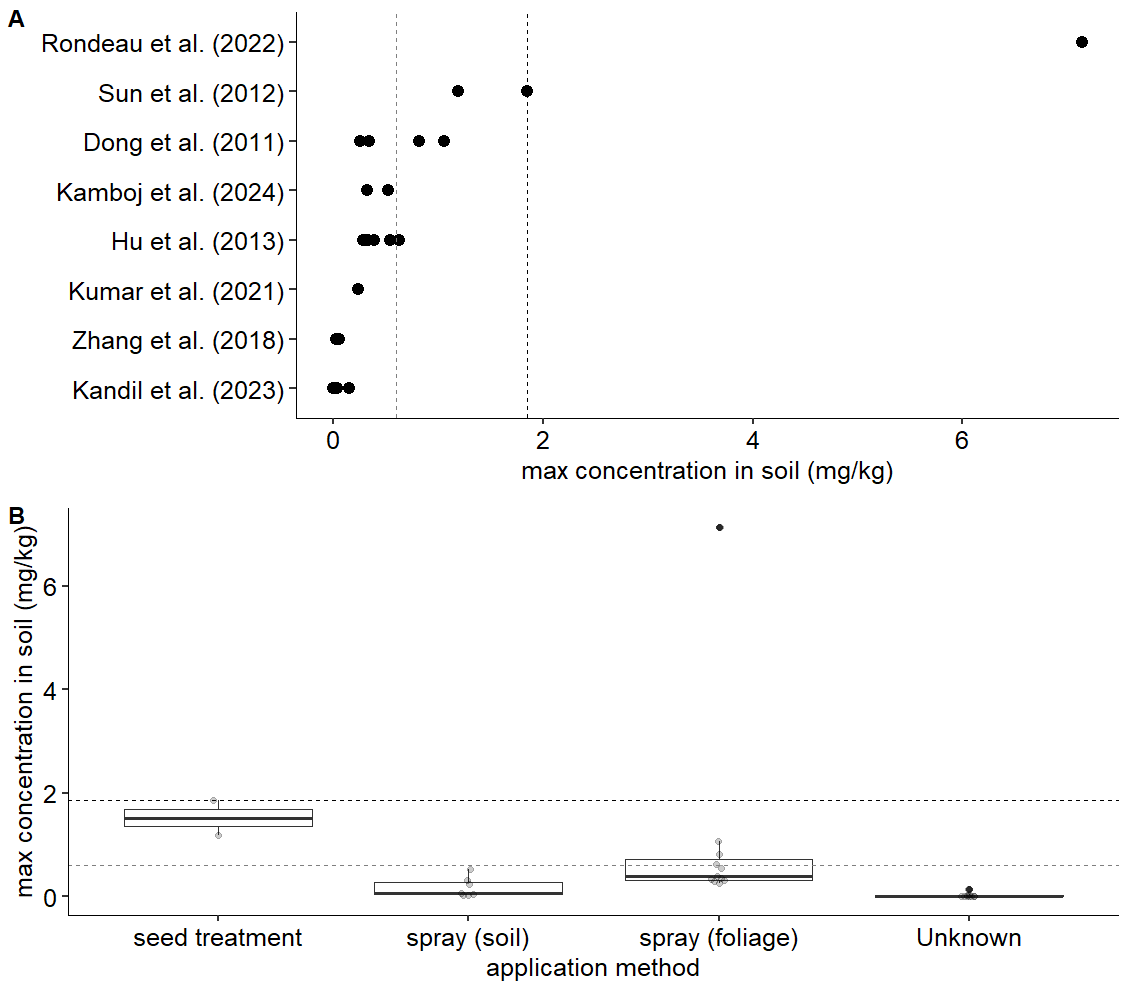


Figure S2: residues of cyantraniliprole measured across literature studies (see Table 1). The grey dashed line represents the best-case exposure level in our study, while the black dashed line represents the worst-case. The highest residue was measured in Kandil et al., 2023. However, the application rate per hectare in this experiment is unknown.

Table S5: the soil exposure data retrieved from the open literature.

| **Reference** | **Application** | **Depth (cm)** | **Crops** | **Application rate (unit)** | **Residue (mg/kg)** | **Notes** |
| --- | --- | --- | --- | --- | --- | --- |
| Zhang et al. (2018) | seed treatment | 15 | maize | 2 (g/kg seed | 1.85 |  |
| Zhang et al. (2018) | seed treatment | 15 | maize | 2 (g/kg seed | 1.19 |  |
| Rondeau et al. (2022) | unknown | 10 | apple | n/a | 0.001 |  |
| Rondeau et al. (2022) | unknown | 10 | apple | n/a | 0.007 |  |
| Rondeau et al. (2022) | unknown | 10 | apple | n/a | 0.001 |  |
| Rondeau et al. (2022) | unknown | 10 | apple | n/a | 0.042 |  |
| Rondeau et al. (2022) | unknown | 10 | apple | n/a | 0 |  |
| Rondeau et al. (2022) | unknown | 10 | apple | n/a | 0.001 |  |
| Rondeau et al. (2022) | unknown | 10 | apple | n/a | 0.03 |  |
| Rondeau et al. (2022) | unknown | 10 | apple | n/a | 0.001 |  |
| Rondeau et al. (2022) | unknown | 10 | apple | n/a | 0.149 |  |
| Rondeau et al. (2022) | unknown | 10 | apple | n/a | 0.004 |  |
| Hu et al. (2013) | spray (bare soil) | 10 | melon | 100 (g/ha) | 0.52 |  |
| Hu et al. (2013) | spray (bare soil) | 10 | melon | 100 (g/ha) | 0.32 |  |
| Sun et al. (2012) | spray (bare soil) | 10 | pak-choi | 120 (g/ha) | 0.03 |  |
| Sun et al. (2012) | spray (bare soil) | 10 | pak-choi | 120 (g/ha) | 0.05 |  |
| Sun et al. (2012) | spray (bare soil) | 10 | pak-choi | 120 (g/ha) | 0.03 |  |
| Sun et al. (2012) | spray (bare soil) | 10 | pak-choi | 120 (g/ha) | 0.06 |  |
| Dong et al. (2011) | spray (bare soil) | 10 | cucumber, tomato | 90 (g/ha) | 0.24 | value estimated from figure |
| Kamboj et al. (2024) | spray (foliage) | 15 | chili | 60 (g/ha) | 0.29 |  |
| Kamboj et al. (2024) | spray (foliage) | 15 | chili | 75 (g/ha) | 0.33 |  |
| Kamboj et al. (2024) | spray (foliage) | 15 | chili | 120 (g/ha) | 0.54 |  |
| Kamboj et al. (2024) | spray (foliage) | 15 | chili | 60 (g/ha) | 0.31 |  |
| Kamboj et al. (2024) | spray (foliage) | 15 | chili | 75 (g/ha) | 0.39 |  |
| Kamboj et al. (2024) | spray (foliage) | 15 | chili | 120 (g/ha) | 0.63 |  |
| Kumar et al. (2021) | spray (foliage) | 15 | cabbage | 60 (g/ha) | 0.256 | 2 (20 - day) interval |
| Kumar et al. (2021) | spray (foliage) | 15 | cabbage | 60 (g/ha) | 0.819 | 2 (20 - day) interval |
| Kumar et al. (2021) | spray (foliage) | 15 | cabbage | 120 (g/ha) | 0.343 | 2 (20 - day) interval |
| Kumar et al. (2021) | spray (foliage) | 15 | cabbage | 120 (g/ha) | 1.061 | 2 (20 - day) interval |
| Kandil et al. (2023) | spray (foliage) | 15 | tomato | 75 (ml/100l) | 7.136 | unknown rate (g/ha) |

# **References**

Boesten, J., Helweg, A., Businelli, M., Bergstrom, L., Schaefer, H., Delmas, A., Kloskowski, R., Walker, A., Travis, K., Smeets, L., Jones, R., Vanderbroeck, V., Jacobsen, O.-S., & Yon, D. (1997). *Soil persistence models and EU registration* [Final Report of the Work of the Soil Modelling Work Group of FOCUS, 630]. FOCUS. https://esdac.jrc.ec.europa.eu/public_path/u891/Soil%20persistence%20models%20and%20EU%20registration%E2%80%99%20%281997%29.pdf

Dong, F., Liu, X., Xu, J., Li, J., Li, Y., Shan, W., ... & Zheng, Y. (2012). Determination of cyantraniliprole and its major metabolite residues in vegetable and soil using ultra‐performance liquid chromatography/tandem mass spectrometry. *Biomedical Chromatography*, *26*(3), 377-383.

Hu, X., Zhang, C., Zhu, Y., Wu, M., Cai, X., Ping, L., & Li, Z. (2013). Determination of residues of cyantraniliprole and its metabolite J9Z38 in watermelon and soil using ultra-performance liquid chromatography/mass spectrometry. *Journal of AOAC International*, *96*(6), 1448-1452.

Kamboj, R., Sharma, S., Mandal, K., & Kang, B. K. (2024). Tandem mass spectrometric analysis of residual persistence, decontamination and dehydration on cyantraniliprole residues in Chilli. *Journal of Food Composition and Analysis*, *125*, 105786.

Kandil, M., Moustafa, M., & Saleh, M. (2023). Dissipation kinetics and degradation products of cyantraniliprole in tomato plants and soil in the open field. *Egyptian Journal of Chemistry*, *66*(12), 483-493.

Kumar, N., Narayanan, N., Banerjee, T., Sharma, R. K., & Gupta, S. (2021). Quantification of field‐incurred residues of cyantraniliprole and IN‐J9Z38 in cabbage/soil using QuEChERS/HPLC‐PDA and dietary risk assessment. *Biomedical Chromatography*, *35*(12), e5213.

Rondeau, S., Baert, N., McArt, S., & Raine, N. E. (2022). Quantifying exposure of bumblebee (Bombus spp.) queens to pesticide residues when hibernating in agricultural soils. *Environmental Pollution*, *309*, 119722.

Sun, J., Feng, N., Tang, C., & Qin, D. (2012). Determination of cyantraniliprole and its major metabolite residues in pakchoi and soil using ultra-performance liquid chromatography–tandem mass spectrometry. *Bulletin of Environmental Contamination and Toxicology*, *89*, 845-852.

Zhang, Z., Xu, C., Ding, J., Zhao, Y., Lin, J., Liu, F., & Mu, W. (2019). Cyantraniliprole seed treatment efficiency against Agrotis ipsilon (Lepidoptera: Noctuidae) and residue concentrations in corn plants and soil. *Pest Management Science*, *75*(5), 1464-1472.
